# Target control based on edge dynamics in complex networks

**DOI:** 10.1038/s41598-020-66524-6

**Published:** 2020-06-19

**Authors:** Furong Lu, Kaikai Yang, Yuhua Qian

**Affiliations:** 1 Institute of Big Data Science and Industry, Shanxi University, Taiyuan, 030006 ShanXi China; 2 School of Computer and Information Technology, Shanxi University, Taiyuan, 030006 ShanXi China

**Keywords:** Applied mathematics, Information technology, Complex networks

## Abstract

In the past decade, the study of the dynamics of complex networks has been a focus of research. In particular, the controllability of complex networks based on the nodal dynamics has received strong attention. As a result, significant theories have been formulated in network control. Target control theory is one of the most important results among these theories. This theory addresses how to select as few input nodes as possible to control the chosen target nodes in a nodal linear dynamic system. However, the research on how to control the target edges in switchboard dynamics, which is a dynamical process defined on the edges, has been lacking. This shortcoming has motivated us to give an effective control scheme for the target edges. Here, we propose the k-travel algorithm to approximately calculate the minimum number of driven edges and driver nodes for a directed tree-like network. For general cases, we put forward a greedy algorithm TEC to approximately calculate the minimum number of driven edges and driver nodes. Analytic calculations show that networks with large assortativity coefficient as well as small average shortest path are efficient in random target edge control, and networks with small clustering coefficient are efficient in local target edge control.

## Introduction

Research on the structure and dynamics of complex networks has attracted much attention in recent years. Traditionally, we assume that the topological structure remains static and then study its influence on the network dynamics^1–6^. However, the topological structure is not necessarily static. One of the relatively new research themes is how dynamic processes affect on the evolution of topological structures. In particular, the controllability of a complex network, which we assess by determining whether the network can be driven from any initial state to any desired state within a finite time, is considered one of the focal research subjects and has been studied massively. Liu *et al*. convert the problem on structural controllability into a graph maximum matching problem^6^ and propose the scheme of calculating the number of minimum driver nodes. Yuan *et al*. later expand the results to undirected networks^7^ and propose the exact controllability theory. Yan *et al*. analyse the observational uncertainty and the energy required for control and achieve a series of fundamental yet important theories^8^. Profái applies structural controllability to temporal networks^9^. Additionally, Gu *et al*. study the application of network controllability to brain networks^10^. These problems address how to control the whole system efficiently. However, for certain large systems, we only need to control some target nodes of the network that are associated with specific tasks. Thus, Gao put forward an approximate algorithm to calculate the fewest driver nodes that can be used to control the target nodes, which effectively reduces the usage of driver nodes^11^. Zhang *et al*. make improvements to this algorithm^12^. The above theory pertains to the dynamic process on the nodes.

In natural or artificial systems, some dynamical processes take place on the edges, such as with power grids, in which power generators, transformers and power consumption are the nodes and transmission lines are the edges. The transformers receive power from high-voltage lines and output the lower-voltage power to the consumers along the transmission lines. The power load on a line can be regarded as the state of the edge and it constantly changes during the process of power transmission.

Subsequently, researchers have made progress modeling the dynamics on the edges of a network. Tamas Nepusz *et al*. study the formation of an edge dynamics system and analyse the structural controllability of edge dynamics^13^, which transforms the edge dynamics in the original networks into the nodal dynamics in the corresponding linear graph. An efficient solution is proposed to compute the minimum driven edges and driver nodes.

However, in many systems, it is inefficient or unnecessary to address all edges. In a power grid, instead of controlling all the lines, we sometimes need to control specified lines to avoid outage on power lines due to overloads. Generally, We may also seek to calculate the minimum driven edges and driver nodes to control the target edges in the edge dynamics, which can be transformed into calculating the minimum driver nodes to control the target nodes in the corresponding line graph. However, the calculation process is time-consuming. In the whole system, the nodes carrying input signals are called driver nodes, the edges linked to the driver nodes are called driven edges. Pang *et al*. generalize the edge dynamics to undirected networks and show a series of results^14–16^. Moreover, they explore the edge target control problem from the perspective of driver nodes^17^.

In this paper, we present a greedy algorithm to solve the problem of target edges’ control and focus on the optimization of the number of driven edges. First, we select the target edges in two different ways: randomly and locally. In the first scheme, we randomly select target edges at random according to the specified probability $p$. In the other scheme, we select target edges from connected components in a depth-first strategy. For a specific tree-like network, we propose a k-travel algorithm to obtain the minimum number of drivers to control the collection of target edges. For the general case, we also give an approximate algorithm TEC to calculate the minimum number of driven edges and driver nodes to control the states of target edges. Finally, we analyse the influence of the topology on the control efficiency.

To evaluate the efficiency of our algorithm, we apply the following metric:

$$\varepsilon =0.5-{\int }_{0}^{1}(\alpha (f))df,$$

which denotes the control efficiencies of the driver nodes (driven edges) in the random and local schemes by ${\varepsilon }_{N}(r)$, ${\varepsilon }_{M}(r)$, ${\varepsilon }_{N}(l)$ and ${\varepsilon }_{M}(l)$. In addition, $\alpha =\frac{{P}_{D}}{{N}_{D}}$, ${P}_{D}$ is the number of driven edges (driver nodes) under target edge control, and ${N}_{D}$ is the number of driven edges (driver nodes) under switchboard dynamic control of the whole network. $f$ denotes the proportion of edges that we selected from the network. When $\varepsilon =0$, the control efficiency is neutral, which means that to control f fraction of edges in networks, we need $f\,\ast \,N$ driver nodes(driven edges). When $\varepsilon \mathrm{ < 0}$ ($\varepsilon \mathrm{ > 0}$), the target edge control is less (more) efficient than the situation with $\varepsilon =0$. The simulation results and analytic calculation show that SF networks with large average degrees are suitable for target edge control, and the local target control obtains a higher control efficiency than the random target control. The control efficiency increases with the average degree of the SF networks and it is higher on driven edges than on driver nodes. In real networks, the results are similar to the cases in artificial networks.

## Method

In the real world, the dynamical processes performed in the vast majority of systems are nonlinear. However, we can not understand the dynamics of many complex systems deeply, such as the neural network of the human brain. Thus, before exploring the dynamical rules in a nonlinear system, it is essential to lucubrate the dynamics of linear systems, as they are the basis of solving the nonlinear problems. Here, we will firstly introduce the linear time-invariant(LTI) system:

1 $$\{\begin{array}{ccc}\dot{x} & = & Ax+Bu,\\ y & = & Cx,\end{array}$$

where $x\in {R}^{N},y\in {R}^{S}$ and $u\in {R}^{M}$ are the state vector, output vector and control inputs, respectively. $A\in {R}^{N\times N},B\in {R}^{N\times M}$ and $C\in {R}^{S\times N}$ represent the transposed matrix of the coupled matrix, the input matrix and the output matrix, respectively. The simplified representation of the above LTI system is $(A,B,C)$. Here, dim($C$) denotes the dimension of the subspace $C$, through which we simplify the dimension of system as $(A,B,C)$ (e.g., $dim(A,B,C)$ is denoted as $d(A,B,C)$).

Generally, the target controllability can be regarded as the output controllability of the LTI system. Hence, we give the following definition and theorem.

### Definition 1

(output controllability)^18^ A system is output controllable if we can move its output from any initial state to any desirable state in a finite time interval with an appropriate input. The LTI system $(A,B,C)$ is output controllable if and only if its output controllability matrix has full rank:

2 $$d(A,B,C)=rank[C(B,AB,{A}^{2}B,\ldots ,{A}^{N-1}B)]=S\mathrm{}.$$

Here, the target edges can be seen as C, A and B are the matrices given by Eq. (1).

Let $G(V;E)$ denote a network, $V$ denote the node set and $E$ denote the edge set. The numbers of nodes and edges are denoted by $N$ and $M$, respectively. $X=[{x}_{1},{x}_{2},\cdot \cdot \cdot ,{x}_{{\rm{M}}}]$ denotes the state vector of each edge, which can be viewed as the packages on a router network or the loads on a line in a power network. ${y}_{{\rm{v}}}^{+}$ and ${y}_{{\rm{v}}}^{-}$ denote the states of the inbound edges and outbound edges of vertex $v$, respectively. Generally, the states of the outbound edges ${y}_{{\rm{v}}}^{+}$ are affected by the states of the inbound edges ${y}_{{\rm{v}}}^{-}$, the losses of inbound edges ${\tau }_{{\rm{v}}}\otimes {{y}_{{\rm{v}}}}^{+}(t)$ and the external disturbance on the corresponding nodes ${\sigma }_{{\rm{i}}}{u}_{{\rm{i}}}(t)$. ${M}_{{\rm{v}}}$ is a switching matrix that denotes the adjacency relationship between the inbound edges and the outbound edges.

The equation of the switchboard dynamic process^13^ is as follows:

3 $${\dot{{y}_{{\rm{v}}}}}^{+}({\rm{t}})={M}_{{\rm{v}}}{y}_{{\rm{v}}}^{-}({\rm{t}})-{\tau }_{v}\otimes {{y}_{{\rm{v}}}}^{+}({\rm{t}})+{\sigma }_{{\rm{i}}}{u}_{{\rm{i}}}({\rm{t}}),$$

where ${\sigma }_{i}$ is 1 if vertex i is a driver node and zero otherwise, and $\otimes$ denotes the entry-wise product of two vectors of the same size.

Here, Eq. (1) can be rewritten in terms of ${x}_{{\rm{i}}}$. The connection of the switchboard dynamic and a standard linear dynamical system can be rewritten in terms of ${x}_{{\rm{i}}}$ as

4 $$\dot{X}=(W-T)X+Hu,$$

where $W=[{w}_{{\rm{kj}}}]$ and ${w}_{{\rm{kj}}}$ may be nonzero if and only if the head of edge ${e}_{{\rm{k}}}$ is the tail of edge ${e}_{{\rm{j}}}$. $T\in {R}^{M\times M}$ is a diagonal matrix with the damping term on the main diagonal. $H$ is a diagonal matrix where the $i$ th diagonal element ${\sigma }_{i}$ is the tail of the $i$ th edge. Furthermore, Eq. (4) can be rewritten in a linear time-invariant dynamical form $\dot{X}=AX+Bu$ with $A=W-T$ and $B=H$. In fact, $W$ is the adjacency matrix of the linear graph $L(G)$ on the original graph $G$. The nodes of $L(G)$ are the edges of $G$, and the edge ${e}_{{\rm{i}}{\rm{j}}}^{{\rm{{\prime} }}}$ of $L(G)$ indicates that the head of edge ${e}_{{\rm{i}}}$ is the tail of edge ${e}_{{\rm{j}}}$.

According to the exact controllability, the damping matrix $T$ with identical diagonal elements has no effect on the edge controllability of the network characterized by $W-T$^14^. However, if the diagonal elements are nonzero, the system can be controlled by only one driver node according to the theory of structural controllability^5^. Except in the above two cases, we need to account for the influence of matrix $T$ on the controllability. The main results of the switchboard dynamics are as follows^13^.

The minimum set of driver nodes required to maintain the structural controllability of the switchboard dynamics on a network $G(V,E)$ is determined by selecting the divergent vertices of G and one arbitrary vertex from each balanced component. The divergent node $v$ is the node with ${d}_{{\rm{v}}}^{-} < {d}_{{\rm{v}}}^{+}$, and the balanced component is the component such that each node satisfies ${d}_{{\rm{v}}}^{+}={d}_{{\rm{v}}}^{-}$. For vertex $v$, ${d}_{{\rm{v}}}^{+}$ is the out-degree and ${d}_{{\rm{v}}}^{-}$ is the in-degree. In addition, ${M}_{{\rm{D}}}$ is the minimum of the driven edges. Then, we have

5 $${M}_{{\rm{D}}}=\mathop{\sum }\limits_{{\rm{i}}\mathrm{=1}}^{N}max({d}_{{\rm{i}}}^{+}-{d}_{{\rm{i}}}^{-}\mathrm{,0)}+\mathop{\sum }\limits_{{\rm{i}}\mathrm{=1}}^{c}{\beta }_{{\rm{i}}}\mathrm{}.$$

here, N is the number of vertexes, c is the number of connected components and ${\beta }_{{\rm{i}}}$ is 1 if the $i$ th connected component is balanced and zero otherwise.

Given a directed network $G(V,E)$ where **|V|** = *N* and $|E|=L$ denote the number of vertexes and edges, if ${a}_{{\rm{ji}}}\ne 0$ is in the matrix $A$, then there is a link from node $i$ to node $j$. A set of target edges with size S may be denoted as $\{{c}_{1},{c}_{2}\mathrm{,...,}{c}_{{\rm{S}}}\}$. The target edges may also be denoted as $\{{e}_{1},{e}_{2},\ldots ,{e}_{{\rm{S}}}\}$. In the switchboard dynamics, the controllability of the target edges is equivalent to the controllability of the corresponding nodes in the linear graph $L(G)$; the linear graph is a graph where we view the edge in the original graph G as a node, and two edges are connected when the head of an edge is the tail of the other edge. Formula $y=Cx$ is the state of the target edges ${x}_{{{\rm{c}}}_{1}},{x}_{{{\rm{c}}}_{2}},\,\mathrm{...,}\,{x}_{{{\rm{c}}}_{{\rm{S}}}}$. Similar to the conclusion in ref. ^11^, the target edge controllability can also be regarded as a special output controllability problem on the linear graph.

For a directed network $G$ with the adjacency matrix of its linear graph: A = [${a}_{{\rm{ij}}}$], ${a}_{{\rm{ij}}}$ = 1 if there is a link $({e}_{{\rm{i}}},{e}_{{\rm{j}}})$ and 0 otherwise.

### Definition 2

(Edge distance) The distance between two edges is the number of internal nodes on a path that connects the two edges, and it is denoted by *d*′.

**Figure 1:**
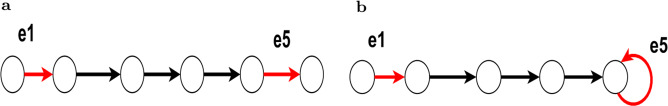
The distance between two edges. The figure shows the distance between two edges along a path. The distance from edge $e1$ to edge $e5$ is four, which is the number of nodes between $e1$ and $e5$.

This definition is used in the k-travel theory, and it differs from the distance between two nodes, which is shown in Fig. 1.

We have ${d}_{{\rm{ij}}}^{\,{\prime} }=k$ if the $(i,j)$ entry of matrix ${A}^{k}$ is nonzero. For general directed networks, ${d}_{{\rm{ij}}}^{\,{\prime} }$ could have multiple values because there could be many different walks connecting edge $i$ and edge $j$. For a directed tree, all of the paths are unique. Consequently, ${d}_{{\rm{ij}}}^{\,{\prime} }$ is single-valued for any edge pair $(i,j)$ in $T$. To clarify algorithm 1, we first introduce several definitions:

### Definition 3

(Structurally equivalent). Two matrices $A=({a}_{{\rm{i}}j})$ and $\tilde{A}=({\tilde{a}}_{{\rm{i}}j})$ with the same size are said to be structurally equivalent if the entries of $A$ being nonzero implies that entries in the corresponding location of $\tilde{A}$ are also nonzero^5^. Moreover, if any entries in $A$ are zeros, the corresponding entry $\tilde{A}$ must also be zero, which is applicable for the structurally equivalence of systems $(A,B,C)$ and $(\tilde{A},\tilde{B},\tilde{C})$.

### Definition 4

(Generic dimension)^19^. The generic dimension $gd(A,B,C)$ of the output state space is defined by

6 $$gd(A,B,C)=\mathop{{\rm{\max }}}\limits_{\tilde{A},\tilde{B},\tilde{C}}rank(\tilde{A},\tilde{B},\tilde{C}),$$

where $\tilde{A},\tilde{B},\tilde{C}$ are structural equivalent to $A,B,C$, respectively. The generic dimension is used to describe the control range of the edge. Based on the switchboard dynamics, we use this k-travel theory to give an effective algorithm to find the controllable set of any edge in the directed tree, especially the root edge.

### Theorem 1

(k-travel theory) For a directed tree, we can control the target edges set ${ {\mathcal L} }_{{\rm{i}}}=\{{e}_{{{\rm{i}}}_{1}},{e}_{{{\rm{i}}}_{2}},\,\mathrm{...,}\,{e}_{{{\rm{i}}}_{{L}_{i}}}\}$ by controlling an edge ${e}_{{\rm{i}}}$ provided that ${d}_{{\rm{ik}}}^{{\prime} }$ (the distance from edge ${e}_{{\rm{i}}}$ to edge ${e}_{{\rm{k}}}$) meets ${d}_{{\rm{ik}}}^{{\prime} }=k-1$ for every integer $k\in \mathrm{[1,}{L}_{{\rm{i}}}]$.

### Proof:

In switchboard dynamics, Eq. (2) can be written in a linear time-invariant dynamical form $\dot{X}=AX+Bu$, where $A$ is the adjacency matrix of the linear graph $L(G)$ of the original graph $G$. The nodes of $L(G)$ denote the edges of $G$, and the edge ${e{\prime} }_{{\rm{ij}}}$ of $L(G)$ indicates that the head of edge ${e}_{{\rm{i}}}$ is the tail of edge ${e}_{{\rm{j}}}$.

According to the output controllability theorem, edge *e*_*i*_ can control all of the edges in ${\mathcal L}$_*i*_ if the generic dimension of the matrix ${\mathcal L} ={l}_{i,\alpha }[{b}_{{\rm{i}}},A{b}_{i},{A}^{2}{b}_{{\rm{i}}},\,\mathrm{...,}\,{A}^{N-1}{b}_{{\rm{i}}}]$ is ${L}_{{\rm{i}}}$, i.e., if

7 $$gd( {\mathcal L} )=gd({l}_{{\rm{i}},\alpha }[{b}_{{\rm{i}}},A{b}_{{\rm{i}}},{A}^{2}{b}_{{\rm{i}}},\ldots ,{A}^{N-1}{b}_{{\rm{i}}}])={L}_{{\rm{i}}},$$

where ${l}_{{\rm{i}},\alpha }=I(\{{i}_{1},{i}_{2},\,\mathrm{...,}\,{i}_{{{\rm{L}}}_{i}}\})$ represents an ${L}_{{\rm{i}}}\times M$ matrix that contains the $\{{i}_{1},{i}_{2},\,\mathrm{...,}\,{i}_{{{\rm{L}}}_{{\rm{i}}}}\}$ th rows of the identity matrix $I$. Moreover, ${b}_{{\rm{i}}}$ is the $i$ th column of the identity matrix. Here, M denotes the number of edges in $G$, and $\{{i}_{1},{i}_{2},\,\mathrm{...,}\,{i}_{{{\rm{L}}}_{{\rm{i}}}}\}$ are the corresponding indices of the edges set ${\mathcal L}$_*i*_ in L(G). The $M\times 1$ vector ${A}^{k}{b}_{{\rm{i}}}$ contains nonzero entries corresponding to those edges with ${d}_{{\rm{ij}}}^{{\prime} }=k$; i.e., the distance between edge *e*_**i** _and ${e}_{{\rm{j}}}$ is $k$ in $G$.

Given a directed-tree network, there is only one edge in ${ {\mathcal L} }_{{\rm{i}}}$ that satisfies ${d}_{{\rm{ij}}}^{{\prime} }=k$, which is assumed to be ${e}_{{\rm{k}}}$ with index ${i}_{{\rm{k}}}$ in $L(G)$. Thus, We have ${l}_{{\rm{i}},\alpha }{A}^{k}{b}_{{\rm{i}}}={\beta }_{{\rm{k}}}{I}_{{i}_{{\rm{k}}}}$, where ${\beta }_{{\rm{k}}}$ is a nonzero constant. ${I}_{{{\rm{i}}}_{{\rm{k}}}}$ is the ${i}_{{\rm{k}}}$ th column in the identity matrix. Hence, we have

8 $$gd( {\mathcal L} )=gd[{\beta }_{1}{I}_{{{\rm{i}}}_{1}},{\beta }_{2}{I}_{{{\rm{i}}}_{2}}\mathrm{,...,}{\beta }_{{L}_{{\rm{i}}}}{I}_{{{\rm{i}}}_{{L}_{i}}}].$$

Since ${I}_{{{\rm{i}}}_{1}},{I}_{{{\rm{i}}}_{2}},\,\mathrm{...,}\,{I}_{{{\rm{i}}}_{{L}_{i}}}$ are independent, $gd( {\mathcal L} )={L}_{{\rm{i}}}$. According to the output controllability theorem, we can control the the edges in ${\mathcal L}$_*i*_ by controlling the edge ${e}_{i}$.

**Figure 2:**
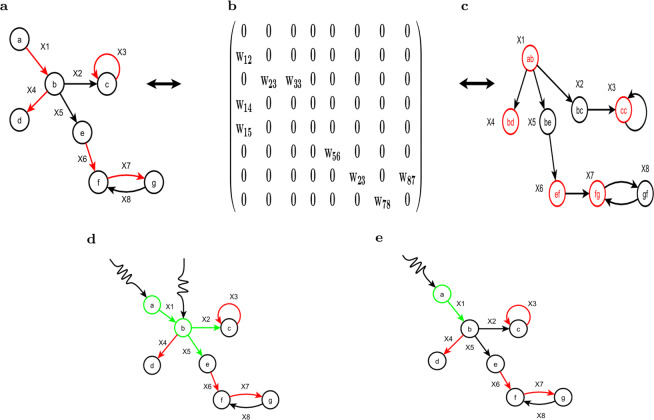
Target edge control based on switchboard dynamics and the k-travel theory. **(a)** The graph is the original network, and the target edges which are indicated in red. **(b)** The switchboard matrix of the original matrix. **(c)** The linear graph of the original network. The nodes in graph(c) are the edges in graph(a). **(d)** The driver nodes and the driven edges that we obtained by using SBD theory. According to the SBD, we need two driver nodes(indicated in green) and three driven edges(indicated in green) to control the whole network. If we want to control the target edges, we need two nodes(a and b) and three driven edges $(x\mathrm{1,}\,x\mathrm{2,}\,x\mathrm{5)}$. **(e)** In fact, we need only one node(*a*) and one driven edge($x1$) to control the states of the target edges, which can be obtained according to the k-travel theory.

Based on the above theorem, we can find the set of edges controlled by edge ${e}_{{\rm{i}}}$ in a directed tree. Figure 2 shows that two driven edges are required to control the target edges under the SBD theory, and k-travel theory requires only one such edge.

In SBD theory, to control all of the edges, the minimum number of driver nodes and driven edges can easily be obtained by checking the in-degree and out-degree of each node.

For the problem of a single edge input, we propose a k-travel theory based on the edge dynamics. Our theory shows that by controlling one edge, we can control a set of target edges given that the lengths from the driven edge to the rest of the target edges are different from each other. We verify that the approach based on k-travel theory is more efficient than the traditional edge dynamics method because it can control more edges according to output controllability theory (Lemma 1).

**Figure 3:**
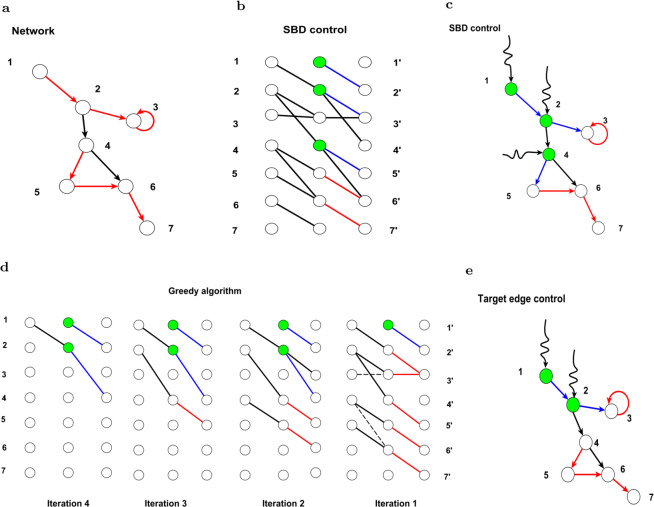
Controlling the states of the edges in a network. **(a)** A directed network with target edges(the red lines). **(b)** We locate the out-edges of the given nodes on its right, and the in-edges on the left. In this way, we construct a bipartite graph. We use SBD theory to obtain the driven edges and the driver nodes, which are marked with blue and green. The driven edges are {(1,2),(2,3),(4,5)} and the driver nodes are {1,2,4}. **(c)** By controlling the three nodes and the three driven edges, all the target edges can be controlled. Moreover, the whole system can be controlled. **(d)** We use the greedy algorithm to control the target edges. In the first iteration, the target edges are shown on the right (shown in red). For the tail node of each target edge, if in_degree(the number of edges on the left) is not less than out_degree(the number of edges on the right), we select the same number of out_edges on the left. Otherwise, the extra target edges are the driven edges according to SBD theory. In the first iteration, the target edge (1,2) is a driven edge(highlighted in blue). In the next iteration, the edges on the left of the bi_layer bipartite graph are the new target edges in the second iteration. After four rounds of iteration, we obtain the driver node 1,2 and the driven edge (1,2),(2,3). **(e)** By controlling the driver node calculated from the TEC, we can control the driven edge. Then, all the targets can be controlled.

Although the method based on k-travel theory is efficiently control tree-like networks with single driven edge (which is shown in Fig. 2), the situation becomes more complex in real networks. For more general cases, we present an iterative algorithm to approximate the minimum number of driven edges and driver nodes. We can prove that the driven edges and driver nodes obtained by our algorithm can control the target edges. This algorithm is based on the k-travel theory, and we call it the TEC (target edge control) algorithm (it is shown in Fig. 3a–e).

The main processes of the TEC algorithm are shown in Fig. 3, and they are based on structural control theory^5,6^ as well as the edge dynamics^13^. **Step 1:** We are given a directed network $G$ with target edges(the red lines). **Step 2:** We construct a bi-layer bipartite graph, locate the head nodes of the target edges in the right part, and find these edges’ tail nodes in the left part. Then, we add the edges which points to the tail nodes of the target edges in graph $G$, and we construct another bipartite graph with the tail nodes of the new edges in the left part such that the right part contains their head nodes. **Step 3:** We use the greedy algorithm for target edges control. In the first iteration, for the tail node of each target edge, if the in_degrees of the middle nodes are not less than their corresponding out_degrees, we select the same number of in_edges. Otherwise, the extra target edges are the driven edges ${E}_{{\rm{t}}}$, and the driver nodes are the tail nodes of ${E}_{{\rm{t}}}$, which are denoted by ${D}_{{\rm{t}}}$. The set of total driven edges is $E=E\cup {E}_{{\rm{t}}}$, with tail nodes $D=D\cup {D}_{{\rm{t}}}$. **Step 4:** In the next iteration, the edges reserved in the left part of the bi-layer bipartite graph are the new target edges in the second iteration. If there is still an edge pointing to the tail nodes of the target edges, go back to step 2 . If not, stop. **Step 5:** By controlling the driver nodes calculated from the TEC, we can control the driven edges and then all of the targets can be controlled. In the above control process, the target edges or nodes are controlled in some chronological order. The process is given in the pseudo-code in the following table.

**Algorithm 1:**
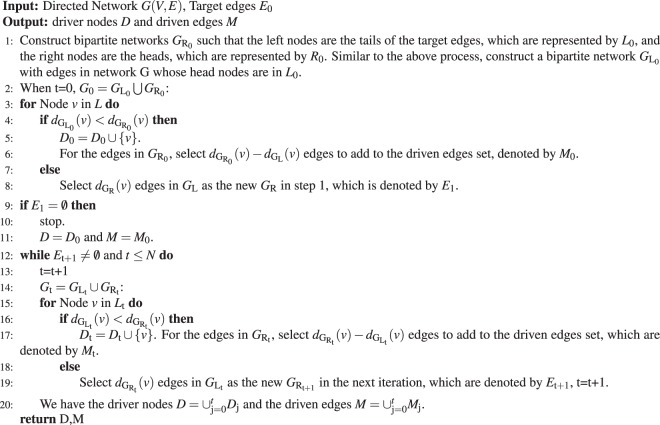
TEC Algorithm.

Here, ${d}_{{{\rm{G}}}_{L}}$(v) means the degree of vertex v in graph ${G}_{{\rm{L}}}$. In each iteration, the times required to calculate the driver nodes and the driven edges are $O(N)$, and the depth of iteration is $N$ at most. Therefore, the complexity of the TEC algorithm is $O({N}^{2})$.

## Result

### Target control of artificial networks

We apply the TEC algorithm to an ER random network and a BA scale-free network (which are denoted by ER network and SF network). The number of vertexes in the ER network is 10000, and the average degree is 6; the result is the average control efficiency over 100 repeated cycles. To quantify the efficiency of the target control for a given fraction $f$, we define the parameter $\alpha ={P}_{{\rm{D}}}/{N}_{{\rm{D}}}$ for both driver nodes and driven edges in two selection schemes. We also use the two schemes to select the target edges, which are random or local. In the local scheme, the target edges are selected from the connected components.

**Figure 4:**
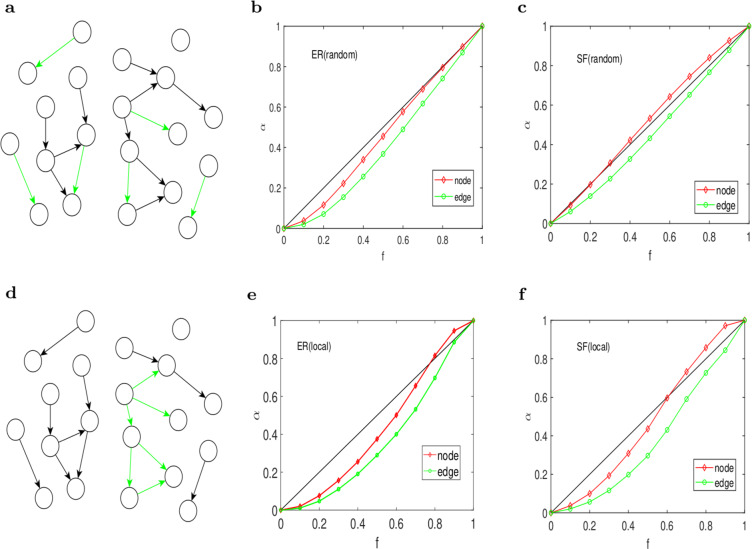
Target edge controllability on the two artificial networks. (**a**) A schematic of the randomly selected edges. **(b)** For the ER network with average degree $\langle k\rangle$ = 6, we show that the normalized fraction $\alpha$ of the driver nodes (highlighted in red) and the driven edges(highlighted in green) varies with the target edges’ fraction in the random scheme. **(c)** For the SF network with average degree $\langle k\rangle$ = 6 and $\gamma =2.4$, we show that the normalized fraction $\alpha$ of the driver nodes and the driven edges varies with the target edges’ fraction in the random scheme. **(d–f)** are the corresponding figures for the local scheme.

Figure 4 shows the two schemes and the results of the driver nodes and driven edges on an ER network and a SF network. The red line represents the ratio of the driver nodes obtained by TEC algorithm to the driver nodes controlling the whole system, and the green line is the corresponding ratio of the driven edges. The black line represents the neutral case that ($\alpha ={P}_{{\rm{D}}}/{N}_{{\rm{D}}}=f$) as a baseline, which means that we need a fraction $f$ of the driven edges(driver nodes) to control the fraction $f$ of the edges. Our algorithm is efficient if the red line is below the baseline. In other words, we use fewer driver nodes or driven edges to control the target edges than with the SBD theory. From Fig. 4b,c, we find that the number of driver nodes obtained from TEC algorithm is less than the corresponding proportion of driver nodes obtained from the SBD theory in the ER network with random scheme. In the SF network, the number of driver nodes under TEC algorithm is slightly more than that obtained under SBD theory framework. However, the number of driven edges in both networks is less, which shows that our algorithm is efficient. In the local scheme(Fig. 4d–f), the number of driver nodes in the two networks is below the baseline when the fraction is small. However, when the fraction is large, the number of driver nodes is above the baseline. In addtion, the control efficiencies of the driven edges in the two networks are below the baseline which means that we can control the target edges by controlling fewer driven edges.

Overall, the efficiency of the driver nodes in the ER network is higher than that of the SF network under the two selection schemes. In the four situations, the metric value of $\varepsilon$ on driven edges are all below the baseline, which shows that our algorithm is efficient in the two artificial networks. Meanwhile, we observe that networks with a big average degree are easy to control and that the homogeneous networks are easier to be controlled than the heterogeneous networks in target edge control.

### Factors of target edge control in artificial networks

**Figure 5:**
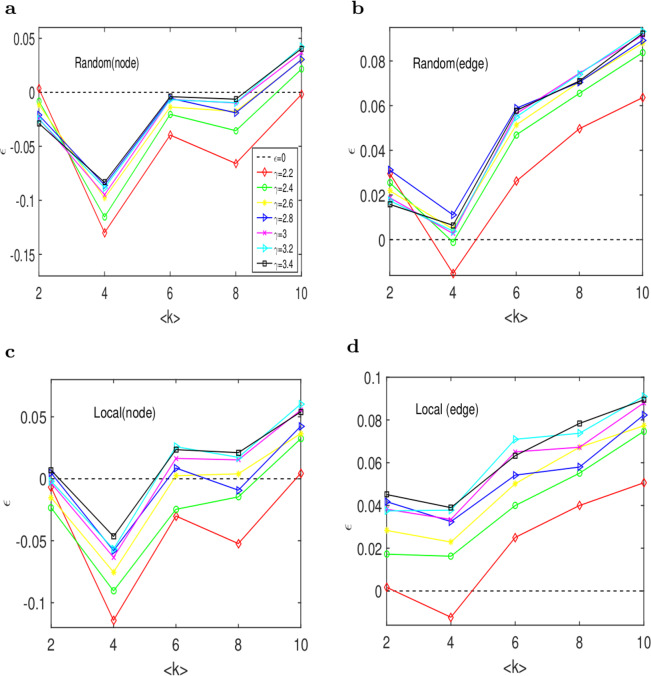
Analysis of the influence factors of the target control efficiencies of SF networks. (**a**) The overall control efficiency $\varepsilon$ on the driver nodes for SF networks varies with the average degree $\langle k\rangle$ for different degree exponents *γ* in the random scheme. **(b)** The overall control efficiency $\varepsilon$ on the driven edges for SF networks varies with the average degree $\langle k\rangle$ for different degree exponents *γ* in the random scheme. **(c)** The overall control efficiency $\varepsilon$ on the driver nodes for SF networks varies with the average degree $\langle k\rangle$ for different degree exponents *γ* in the local scheme. **(d)**The overall control efficiency $\varepsilon$ on the driven edges for SF networks varies with the average degree $\langle k\rangle$ for different degree exponents *γ* in the local scheme. In (a,b), the efficiencies of the driver nodes and the driven edges increase monotonically with the degree exponents. Most of the efficiency of the driver nodes are negative, and they first decrease and then increase. However, the efficiencies of the driven edges are nearly positive, and they have the same trend as the driver nodes. In the local scheme, though we have the same situation as in the random scheme, the local control outperforms the random control.

The above results inspire us to consider which topological characteristics determine the control efficiency. To ask this question, we analyse the effects of average degree and power exponent on the control efficiency. Here, we mainly study the problem on SF networks^20^ with the average degree varying from 2 to 10 at intervals of 2 and with the power index varying from 2.2 to 3.4 in a step 0.2. In addition, we fix the power exponent and the average degree to observe the marginal influence of the two factors on the control efficiency, which is shown in Fig. 5.

Figure 5a,b show that the control efficiency of driver nodes and driven edges first decrease and then increase as the average degree increases in the random scheme. Furthermore, the control efficiency on the driven edges is higher than that of the driver nodes. Figure 5c,d show the results in the local scheme, and we observe a similar trend in the random scheme. The reason is that we consider the edges in network G as the nodes of its linear graph in our algorithm and make use of target control theory based on the nodes. For this reason, our algorithm focuses on the optimization of the driven edges. Moreover, local target edge control is more efficient than random target edge control. In general, when the average degree increases, the control efficiency increases, which can be attributed to the fact that networks gain more connectivity when the average degree is increasing.

### Target control of real networks

We also apply our algorithm to several real networks, where s838, s420 and s208 are three electronic circuit networks^21^ whose average degree is between 1.54 and 1.6. The neuronal network ($C.elegans$)^22^ is a directed weighted network representing the neural network of $C.elegans$ with 297 neurons and 2359 synaptic connections. $TRN$-$Yeast$-1 and $TRN$-$Yeast$-2 are two transcription networks of yeast. $E.\,coli,C.\,elegans$ and $S.\,cerevisiae$^23^ are three metabolic networks. Ythan, Grassland, Little rock and Seagrass are four food webs^24–26^ on a small scale.

From Table 1, the efficiency of the driven edges on the three networks($C.\,elegans$, $TRN$-$Yeast$-1 and $TRN$-$Yeast$-2)^27,28^ is slightly better than that of the driver nodes, which shows that their topological structures are suitable for target edge control in both schemes with respect to the metric. In the metabolic networks ($E.\,coli$, $C.\,elegans$ and $S.\,cerevisiae$), fewer driven edges and driver nodes are needed to control the target edges in the two schemes. Our method does not optimize the control efficiency of the four food webs under both schemes. The efficiency of the driven edges in the random scheme is higher than that in the local scheme. Conversely, we employ more driver nodes in random control than in local control. In random target edge control, the control efficiencies of the driver nodes of most networks are negative except the metabolic networks, which implies that most networks do not perform well in terms of the driver nodes in the random scheme. In term of the efficiency of driven edges in the random scheme, the neuronal network, the regulatory networks and the metabolic networks surpass those of the other networks, which means that we need fewer driven edges to control the target edges under the TEC algorithm than that the SBD theory would require on these networks. With reagard to controlling the target edges in local scheme, the efficiencies of the driver nodes is high in the electronic circuit networks and the metabolic networks. For the driven edges in the local scheme, we select target edges in a local manner, the efficiency is satisfactory in all the networks except for the food web.

**Table 1:** The topological characters and the efficiencies of the real networks.

	Nodes	Edges	$\langle k\rangle$	${N}_{{\rm{D}}}$	${M}_{{\rm{D}}}$	${\varepsilon }_{{\rm{N}}}(r)$	${\varepsilon }_{{\rm{M}}}(r)$	${\varepsilon }_{{\rm{N}}}(l)$	${\varepsilon }_{{\rm{M}}}(l)$
s838	512	819	1.6	0.223	0.350	−0.085	−0.042	0.108	0.093
s420	252	399	1.58	0.25	0.348	−0.078	−0.049	0.102	0.078
s208	122	189	1.549	0.246	0.344	−0.091	−0.066	0.087	0.062
$C.\,elegance$	297	2359	7.895	0.549	0.377	−0.071	0.05	−0.024	0.085
TRN-Yeast-1	4441	12873	2.899	0.0336	0.964	−0.424	0.007	−0.229	0.002
TRN-Yeast-2	688	1079	1.568	0.1773	0.952	−0.225	0.0003	−0.097	0.004
$E.\,coli$	2275	5763	5.07	0.182	0.121	0.0279	0.0789	0.1403	0.1715
$C.\,elegance$	1173	2864	2.442	0.182	0.1156	0.0127	0.0776	0.1824	0.2035
$S.\,cerevisiae$	1511	3833	2.54	0.185	0.1158	0.0134	0.0791	0.1475	0.1838
Ythan	135	601	4.452	0.3037	0.597	−0.1515	0.0331	−0.2037	−0.0093
Grassland	88	137	1.557	0.3182	0.606	−0.1042	−0.0041	−0.0500	−0.0247
Little rock	183	2494	13.63	0.6393	0.603	−0.2177	−0.0412	−0.1292	0.0482
Seagrass	49	226	4.571	0.4490	0.518	−0.1295	−0.0383	−0.1182	−0.0517

We test the greedy algorithm on the real networks and list the following metrics: the average degree $\langle k\rangle$, where D represents the diameter of the network, ASP is the average shortest path, $N\_{\rm{D}}$ denotes the efficiency of the driver nodes in the full control in the switchboard dynamic scheme(SBD), $M\_{\rm{D}}$ show the efficiencies of the driven edges, ${\varepsilon }_{{\rm{N}}}(r)$, ${\varepsilon }_{{\rm{M}}}(r)$ are the similar metrics in the random target edges’ control, and ${\varepsilon }_{{\rm{N}}}(l)$, ${\varepsilon }_{{\rm{M}}}(l)$ represent the corresponding metrics in the local scheme.

In general, we can see that the same types of networks present similar results. The performance of different network types is quite different, but why are the results so different? Is there any correlation between the control efficiency and the topological character? In artificial networks, we see that the control efficiency of SF networks is closely related to the average degree and the degree exponent. Does the same hold for real networks? We show the topological characteristics of these networks in Table 2.

**Table 2:** The topological characters of the real networks.

	N	M	AD	D	ASP	CC	AC	H
s838	512	819	1.6	20	8.924	0.027	−0.03	1.2595
s420	252	399	1.58	16	7.282	0.028	−0.0059	1.2347
s208	122	189	1.549	14	6.032	0.03	−0.0020	1.2159
*C.elegance*	297	2359	7.895	14	3.992	0.169	−0.1520	1.8008
TRN-Yeast-1	4441	12873	2.899	17	4.785	0.046	−0.5968	15.4315
TRN-Yeast-2	688	1079	1.568	5	1.441	0.024	−0.4001	4.2544
$E.\,coli$	2275	5763	5.07	17	5.657	0	−0.1617	8.2345
$C.\,elegans$	1173	2864	2.442	30	5.945	0	−0.1733	4.8075
$S.\,cerevisiae$	1511	3833	2.54	22	5.793	0	−0.1812	5.8141
Ythan	135	601	4.452	4	2.151	0.109	−0.2217	2.1194
Grassland	88	137	1.557	3	1.736	0.174	−0.2157	1.7582
Little rock	183	2494	13.63	6	1.898	0.17	−0.2374	1.6148
Seagrass	49	226	4.571	4	1.816	0.132	0.1119	1.2612

N and M are the total numbers of nodes and links, respectively. AD denotes the average degree of the network, D is the network diameter, and ASP denotes the average shortest path. CC and AC are the clustering coefficient and assortativity coefficient, respectively. Nodes with degree 1 are excluded from the calculation of the clustering coefficient. H is the degree heterogeneity, which is defined as H = $\frac{\langle {k}^{2}\rangle }{{\langle k\rangle }^{2}}$.

We list 6 topological features of the networks, including their average degree(AD), diameter(D), average shortest path(ASP), clustering coefficient(CC), assortativity coefficient(AC), and heterogeneity(H). From the table, we observe that the electronic circuit networks, the metabolic networks and the transcriptional regulatory network have larger diameter. However, the electronic circuit networks have smaller degrees, longer average shortest paths, and more obvious heterogeneity. The food webs have greater average degrees, smaller diameters as well as higher clustering coefficient.

To find the relationship between the topological characteristics and the control efficiency, we take the topology parameters of each network and the corresponding control efficiency index values as samples and calculate the correlation coefficient of each pair of indicators. The D and the ASP are related to the network scale and the average degree, respectively. To facilitate a uniform comparison, we normalize D and ASP by dividing these factors by the network scale *N*. The results are presented in Table 3.

**Table 3:** Pearson’s correlation coefficient.

	${\varepsilon }_{N}(r)$	${\varepsilon }_{M}(r)$	${\varepsilon }_{N}(l)$	${\varepsilon }_{M}(l)$
$\langle k\rangle$	−0.1461	0.0365	−0.3435	−0.0363
D′	0.1787	−0.6473	0.1361	−0.2349
ASP′	0.1279	**−0.7026**	**0.0626**	**−0.3579**
CC	−0.2585	−0.2455	**−0.5988**	**−0.6082**
AC	**0.6599**	**−0.3197**	**0.4930**	**0.1228**
H	−0.4407	0.4252	−0.1837	0.1469

We test the TEC algorithm on the real networks and list the following metrics. The bold numbers are the relatively large values of the correlation coefficient.

As observed from the table, although AC is the dominant factor for ${\varepsilon }_{N}({\rm{r}})$ and ASP is dominant for ${\varepsilon }_{N}({\rm{l}})$, in general, CC is the most influential factor for the control efficiency in local scheme. AC and ASP′ are the main factors in the random scheme. In general, we can conclude that D′, ASP′ and CC are weakly negative correlated to the control efficiency. Form k-travel theory, while the distances from every target edge to the root edge are distinct, then we can control the target edges by solely driving the root edge. However, we can also easily control the whole network. Therefore, our algorithm is not very efficient for target edge control relative to the SBD theory. As a result, the control efficiency is not high. CC is also negatively correlated to the control efficiency. When the clustering coefficient is high, the number of driven edges in target control is not siginficantly different from the corresponding number in SBD theory. Furthermore, the control efficiency is nearly high when AC is high. In Fig. 5(a–d), we observe that the control efficiency at $\langle k\rangle \mathrm{=4}$ is lower than the corresponding efficiency in the other cases. Because the diameter and ASP of SF network at $\langle k\rangle \mathrm{=4}$ are higher than in the other cases, the network’s CC is nearly the same, which is shown in Table 4.

**Table 4:** The topological indices of SF networks.

	$\langle k\rangle =2$	$\langle k\rangle =4$	$\langle k\rangle =6$	$\langle k\rangle =8$	$\langle k\rangle =10$
SF2-2	12 4.519 0	**15** **4.452** **0.078**	13 4.13 0.057	10 3.618 0.05	9 3.567 0.062
SF2-4	13 4.318 0	**18** **5.697** **0.014**	15 4.903 0.015	11 4.375 0.017	10 4.075 0.019
SF 2-6	15 4.99 0	**20** **6.429** **0.007**	15 5.314 0.008	13 4.77 0.008	10 4.386 0.009
SF2-8	13 4.397 0	**22** **7.185** **0.002**	16 5.713 0.004	12 5.003 0.005	11 4.578 0.005
SF3	12 4.329 0	**23** **7**.**479** **0.002**	18 5.96 0.003	13 5.200 0.003	12 4.712 0.004
SF3-2	14 4.802 0	**26** **7.934** **0.002**	17 6.189 0.002	14 5.37 0.002	11 4.835 0.003

We list the different SF networks with different power exponents and average degrees and their three topological indices: D(diameter), ASP(average shortest path), and CC(clustering coefficient).

## Discussion

In this paper, we study the edge target controllability of complex networks. For a directed tree-like network, to find the lowest driver nodes or driven edges needed to control the target edges, we propose a new method: k-travel theory. Base on the situation with multiple inputs, we develop a TEC greedy algorithm to approximately identify the set of the minimum driven edges and driver nodes to control some target edges. Our algorithm is more efficient than the SBD theory in most cases. We adopt the local scheme and the random scheme to study the impact of the network topology on the final results. We also approximately give the minimal drive nodes and driven edges on the artificial and real networks. The results show that our method is efficient in most networks, especially under the local scheme. Moreover, we analyse the topological factors that affect target edge control.

In addition, when the nodal target controllability is based on the structural controllability, our method outperforms the nodal target controllability approach in terms of the computational efficiency with respect to identifying the minimum set of drivers. For example, in each iteration of the TEC algorithm, finding the driver nodes based on the degree-checking requires a run-time with the order of magnitude $O(N)$, whereas the nodal target controllability framework requires a run-time that is $O({N}^{\mathrm{1/2}}L)$ to find the unmatched nodes, where ***L*** is the total number of links.

Our research leads to several questions. Recently, the theory of structural controllability has been extended to temporal networks^9,29^ and multi-layer networks^30^. Extending edge target controllability theory to temporal networks and multi-layer networks may be necessary to combine their own characters. Solving these problems will help further enrich the theory of edge target control.
